# Neonatal Sepsis: Pathogens, Clinical Challenges, and Emerging Solutions

**DOI:** 10.7759/cureus.104238

**Published:** 2026-02-25

**Authors:** Matthew Green, Jyotsna Chawla, Cyril Blavo

**Affiliations:** 1 Foundational Sciences, Nova Southeastern University Dr. Kiran C. Patel College of Osteopathic Medicine, Clearwater, USA

**Keywords:** antibiotic therapy, early-onset sepsis, escherichia coli, global health, group b streptococcus, late-onset sepsis, listeria monocytogenes, neonatal infection control

## Abstract

Neonatal sepsis remains a major cause of preventable newborn deaths worldwide despite advances in maternal screening, antibiotic use, and neonatal care. Persistent diagnostic delays, rising antimicrobial resistance (AMR), and unequal access to resources highlight the urgent need for new approaches. This review summarizes the primary pathogens responsible for early- and late-onset neonatal sepsis, along with their transmission routes, virulence factors, and clinical presentations. We examine promising innovations in rapid molecular diagnostics, next-generation sequencing, artificial intelligence (AI), maternal vaccination, and immunotherapies that have the potential to transform prevention and treatment strategies. By consolidating current evidence and addressing gaps in global care, this review calls for standardized protocols and equitable implementation of these advancements to reduce the global burden of neonatal sepsis.

## Introduction and background

Sepsis has historically been defined as an inflammatory response to infection measured using systemic inflammatory response syndrome (SIRS) criteria [1]. In adults, SIRS is defined as suspected or confirmed infection plus two or more of the following: temperature >38.5°C (101.3°F) or <36.0°C (96.8°F), heart rate >90 beats per minute, respiratory rate >22 breaths per minute or PaCO₂ <32 mmHg, and a white blood cell count >12,000/mm³, <4,000/mm³, or >10% immature (band) forms [2]. However, these fixed physiologic thresholds are not directly applicable to neonates due to age-specific variations in baseline heart rate, respiratory rate, and immune response. Moreover, there is no single universally accepted set of neonatal SIRS or sepsis criteria. In neonatal practice, diagnosis relies on clinical assessment, maternal and perinatal risk factors, laboratory findings, and microbiologic evaluation rather than strict fulfillment of adult SIRS cutoffs. Bacteremia, the presence of bacteria in the blood, may occur with or without symptoms, while “neonate” refers to the first 28 days of life.

Neonatal sepsis is primarily caused by bacterial pathogens but can also result from viral, fungal, or parasitic infections. Transmission can occur in utero, perinatally, or postnatally, and sepsis is classified based on onset timing. Early-onset sepsis (EOS) presents within the first 72 hours of life [3]. Late-onset sepsis (LOS) occurs after this window [3]. Clinical signs are often nonspecific, including lethargy, temperature instability, respiratory distress, and poor feeding, and a high index of suspicion is essential. Blood cultures remain the gold standard for diagnosis, though delays in results necessitate early empiric treatment.

**Figure 1:**
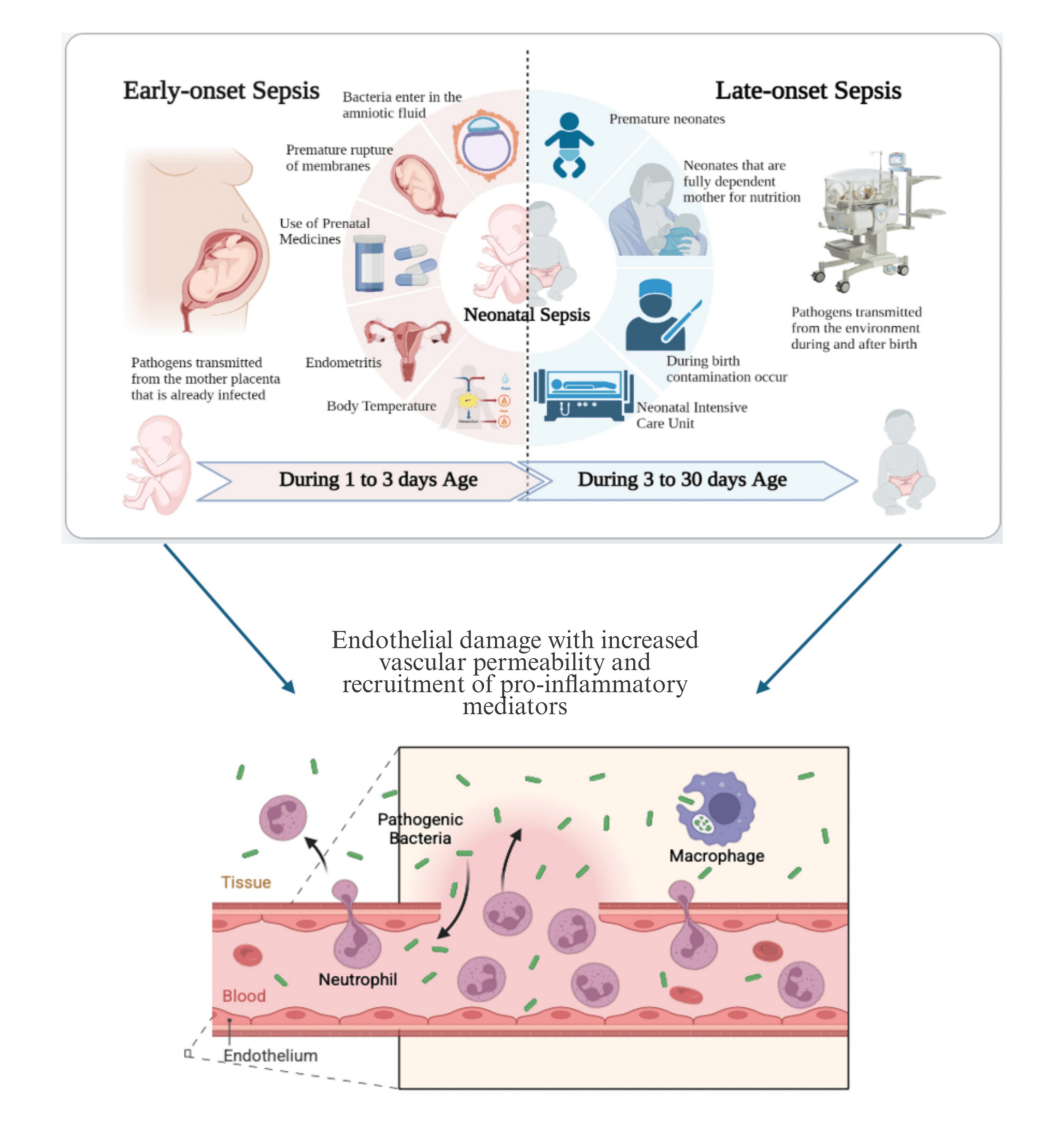
Early- and late-onset neonatal sepsis and associated pathophysiological mechanisms The figure was created by the authors with BioRender.com.

Risk factors of EOS include premature rupture of the membrane, prolonged labor, maternal fever, prematurity, low birth weight, and resuscitation at birth [4, 5]. In contrast, LOS is often linked to invasive procedures, prolonged hospitalization, and healthcare-associated exposures [6]. The most common bacterial causes of neonatal sepsis are Group B Streptococcus (GBS) or *Escherichia coli* (especially *Escherichia coli*, K1 strain), *Listeria monocytogenes*, and *Staphylococcus aureus* [7, 8]. The clinical spectrum and underlying pathophysiology of neonatal sepsis, including differences between early-onset and late-onset presentations, are illustrated in Figure 1.

Globally, neonatal sepsis and related infections affected an estimated 3.88 million neonates in 2021, resulting in over 200,000 deaths [9, 10]. The burden is particularly heavy in low- and middle-income countries (LMICs), where access to screening, antibiotics, and neonatal intensive care is limited. While GBS predominates in upper-middle- and high-income countries (e.g., USA), *Escherichia coli* and *Staphylococcus aureus* are more frequently encountered in LMICs [11]. Mortality rates remain high for many organisms: *Klebsiella pneumoniae* (21.5%), GBS (21.1%), *Listeria monocytogenes* (14.9%), *Escherichia coli* (12.8%), and *Staphylococcus aureus* (11.1%) [12]. These infections may lead to severe complications such as meningitis, which carries a heightened risk of neurodevelopmental impairment or death. Notably, GBS is the leading cause of nonfatal bacterial neonatal meningitis, while *Escherichia coli* and *Listeria monocytogenes* are more common in fatal cases [13].

Given the overlapping clinical manifestations and challenges in diagnosis, a comprehensive understanding of these pathogens and their associated risk factors is critical for effective management and prevention of neonatal sepsis. This review provides a detailed overview of the key pathogens involved in neonatal sepsis, their clinical presentations, virulence mechanisms, and current evidence-based strategies for diagnosis, prevention, and treatment. It also addresses gaps in existing protocols and outlines innovations in diagnostics, immunotherapies, and global health policy aimed at reducing neonatal mortality.

## Review

Key pathogens of neonatal sepsis

Group B Streptococcus (Streptococcus agalactiae)

GBS is the leading cause of EOS in the United States, particularly in term neonates. A Gram-positive, encapsulated, beta-hemolytic coccus, GBS has 10 serotypes (Ia, Ib, II, III, IV, V, VI, VII, VIII, IX), with Ia, II, III, and V predominating in neonatal and maternal infections [14-16]. Colonizing the gastrointestinal and genitourinary tracts of 10% to 30% of pregnant women, as well as amniotic fluid and neonatal respiratory tracts, GBS employs hemolysin, a cytolytic exotoxin that forms pores in host cell membranes, and hyaluronidase, which degrades extracellular matrix hyaluronic acid, to facilitate placental invasion and immune evasion [17-19]. Neonates present with temperature instability, respiratory distress, lethargy, and poor feeding within 24-48 hours [20]. Diagnosis relies on blood cultures, with polymerase chain reaction (PCR) offering rapid detection [20]. Empiric treatment involves administering intravenous ampicillin and gentamicin for seven to 10 days, with the duration extended to 14-21 days for meningitis, as adjusted based on culture sensitivities [21]. Prevention includes universal screening via recto-vaginal swabs at 35-37 weeks’ gestation, followed by intrapartum antibiotic prophylaxis (IAP) with intravenous penicillin or ampicillin, or a risk-based protocol targeting factors like preterm birth or maternal fever [22]. A study by Hasperhoven et al. found no significant difference in GBS incidence between these approaches [22]. The World Health Organization recommends IAP for at least 4 hours pre-delivery, with cephalosporins for penicillin-allergic patients, to minimize resistance risks [23].

Escherichia coli 

*Escherichia coli*, the second most common cause of neonatal sepsis, is prevalent in preterm infants [24]. This Gram-negative, rod-shaped, facultative anaerobe has multiple serotypes, with the K1 capsular strain most associated with sepsis [15]. Its virulence factors, such as hemolysin, which disrupts cell membranes, and fimbriae, which enable adhesion to intestinal, vaginal, and amniotic tissues, facilitate colonization [25]. Clinical features mirror GBS sepsis, including respiratory distress and lethargy [20]. Blood cultures and PCR are diagnostic, with urine and cerebrospinal fluid (CSF) cultures in specific cases [20]. Empiric therapy uses intravenous ampicillin and gentamicin, with cefotaxime for ampicillin-resistant cases, typically for seven to 10 days [26]. Screening for *Escherichia* *coli* differs from GBS and is not universally performed in the United States. In certain countries, it is conducted at 35-37 weeks’ gestation if GBS screening is negative, involving vaginal and rectal swabs followed by IAP with ampicillin, amoxicillin-clavulanate, or cefazolin [22,24]. Prompt blood culture collection before antibiotic administration ensures accurate diagnosis.

Listeria monocytogenes 

*Listeria monocytogenes,* a Gram-positive, motile, facultative intracellular bacillus, causes severe but less common neonatal sepsis, often linked to foodborne outbreaks (e.g., unpasteurized dairy, deli meats) [27]. It is 20 times more prevalent in pregnant women [28]. Virulence factors include internalin, which binds placental E-cadherin for colonization, listeriolysin O, which escapes host cell vacuoles, and ActA, which promotes cell-to-cell spread [29-31]. Neonates present with respiratory distress, fever, poor feeding, jaundice, and, from one study, in 50% of cases with confirmed listeriosis, a maculopapular rash (granulomatosis infantisepticum) was observed [32]. Diagnosis uses blood cultures and PCR, with CSF analysis for meningitis [20]. Empiric therapy with intravenous ampicillin and gentamicin for 7-10 days is typically sufficient [22, 32, 33]. Prevention relies on the Centers for Disease Control and Prevention (CDC) and American College of Obstetricians and Gynecologists (ACOG) guidelines advising pregnant women to avoid high-risk foods, with no routine maternal screening but prompt management of maternal symptoms like fever or nausea [33].

Staphylococcus aureus

*Staphylococcus aureus*, a Gram-positive, coagulase-positive coccus, is a major LOS cause, often nosocomially acquired via hospital equipment like catheters [15, 34]. Subtypes include methicillin-sensitive (methicillin-sensitive *Staphylococcus aureus* (MSSA)) and methicillin-resistant (methicillin-resistant *Staphylococcus aureus* (MRSA)) strains [15]. Virulence factors, including hemolysin, hyaluronidase, and alpha toxin that forms membrane pores, enable tissue invasion [30]. Symptoms include temperature instability, respiratory distress, and irritability in hospitalized neonates [34]. Diagnosis involves blood, urine, and CSF cultures, supplemented by PCR [20]. Empiric therapy for suspected *Staphylococcus aureus* infections in neonates often uses IV ampicillin and gentamicin, with vancomycin or narrow-spectrum penicillins added based on local antibiograms [21,34-36]. In the United States, first-line treatment for MSSA typically includes cloxacillin, oxacillin, or cefazolin, all with similar efficacy. In many parts of Europe, Asia, and Australia, flucloxacillin is the preferred first-line agent for MSSA infections. Both the United States and Europe use vancomycin as first-line therapy for MRSA infections. The CDC recommends MRSA screening in neonatal intensive care units (NICUs) by swabbing the anterior nares, combined with strict hand hygiene and judicious catheter use [37].

GBS, *Escherichia coli, Listeria* ​​​​​​*monocytogenes,* and *Staphylococcus aureus* are the principal pathogens implicated in neonatal sepsis, often presenting with overlapping symptoms such as fever, respiratory distress, lethargy, and poor feeding. Due to the nonspecific nature of these symptoms, timely diagnosis through blood cultures and PCR is essential, followed by empiric therapy, commonly intravenous ampicillin and gentamicin. Effective prevention strategies differ by pathogen: maternal screening and IAP for GBS and *Escherichia* ​​​​​​*coli*, dietary precautions and food safety for *Listeria* *monocytogenes*, and rigorous infection control practices in neonatal intensive care units for *Staphylococcus aureus*. Despite established protocols, challenges such as antibiotic resistance and inconsistencies in global screening guidelines persist. This underscores the need for standardized diagnostic algorithms and vaccine development to reduce neonatal morbidity and mortality. A comparative overview of each pathogen’s transmission, virulence factors, tissue tropism, and clinical features is detailed in Table 1.

**Table 1:** Comparative Profile of Key Pathogens in Neonatal Sepsis Mortality rates adapted from [38]. GI: gastrointestinal; GU: genitourinal; PCR: polymerase chain reaction; EOS: early-onset sepsis; LOS: late-onset sepsis

Variables	Group B Streptococcus	*Escherichia coli*	Listeria monocytogenes	Staphylococcus aureus
Transmission	Maternal membrane rupture	Vertical transmission	Fecal-oral and placental, foodborne outbreaks	Nonsocomial via hospital equipment
Virulence factors	Hemolysin; hyaluronidase	K1 capsule; hemolysin; fimbriae	Internalin surface protein, listeriolysin O, ActA	Hemolysin, hyaluronidase, alpha toxin
Tropism	GI, GU tract of women; upper respiratory tract of newborns	GI tract, vaginal flora, amniotic fluid, CSF	GI, placenta	Human skin, mucosal membranes
Mechanism of invasion	Virulence factors to invade the placenta	Virulence factors to invade the amniotic fluid	Virulence factors to invade the placental tissue	Virulence factors to invade human skin/membranes
CNS involvement	Yes	Yes	Yes	Yes
Major clinical features	Fever, respiratory distress, lethargy, poor feeding, temperature instability	Fever, respiratory distress, lethargy, poor feeding, temperature instability	Fever, respiratory distress, jaundice; occasionally, with a rash (granulomatosis infantiseptica).	Fever, irritability, poor feeding, respiratory distress
Prevention	GBS screening at 35-37 weeks’ gestation	Prepartal vaginal, rectal, or amniotic swabs	Maternal avoidance of unpasteurized dairy and meats. Monitor maternal health	Handwashing; cleaning of hospital equipment
Diagnostic strategy	Blood culture, PCR, and clinical judgement	Blood culture, PCR, and clinical judgement	Blood culture, PCR, and clinical judgement	Blood culture, PCR, and clinical judgement
Global prevalence	23.3%	18.0%	2.2%	9.8% (EOS), 15.5% (LOS)
Mortality rate	21.1%	12.8%	14.9%	11.1%

Therapeutic approaches and emerging solutions

Antibiotic Therapy and Stewardship

Antibiotics remain the first-line treatment for neonatal sepsis, with empirical regimens often including ampicillin and gentamicin for EOS and expanded coverage (e.g., vancomycin and gentamicin) for LOS. Rising antimicrobial resistance (AMR) necessitates stewardship to optimize therapy and minimize microbiome disruption. Real-time antibiograms guide empiric therapy by tracking local resistance patterns. Flannery et al. reported that 10.1% of *Escherichia* ​​​​​​*coli* EOS cases and 66.8% of NICU cases were resistant to ampicillin-gentamicin and ampicillin alone, respectively [39]. Stewardship programs promote evidence-based antibiotic use, with future integration of rapid diagnostics to refine selection and reduce overuse. As shown in Table 2, the first-line antibiotics and second-line options vary by the infectious agent.

**Table 2:** Recommended First-Line and Second-Line Antibiotic Regimens for Neonatal Sepsis by Pathogen The table presents empirical and targeted antibiotic options for common neonatal pathogens, including *Streptococcus agalactiae*, *Escherichia coli*, *Listeria monocytogenes*, and *Staphylococcus aureus*, with adjustments for methicillin-sensitive *Staphylococcus aureus* (MSSA) and methicillin-resistant *Staphylococcus aureus* (MRSA) cases.

Etiologic Agent	First-Line Antibiotics	Second-Line Antibiotics
*Streptococcus agalactiae* (Group B Streptococcus)	Ampicillin + Gentamicin	Penicillin + Gentamicin
Escherichia coli	Ampicillin or Cefotaxime + Gentamicin	Amikacin with Gentamicin resistance
Listeria monocytogenes	Ampicillin + Gentamicin	Vancomycin
Staphylococcus aureus	Ampicillin + Gentamicin. Cloxacillin for MSSA; Vancomycin for MRSA	Linezolid for MRSA

Immunotherapies

Immunotherapies such as intravenous immunoglobulin (IVIG) and granulocyte-macrophage colony-stimulating factor (GM-CSF) are being explored as adjunct treatments for neonatal sepsis [40, 41]. IVIG provides passive immunity by delivering antibodies to neonates with immature immune systems. However, its clinical efficacy remains inconsistent. A large Cochrane review study found that IVIG did not significantly reduce mortality or the incidence of severe infections in neonates [40]. Conversely, smaller studies in preterm or low-birth-weight infants reported a modest reduction in mortality rates [42]. These conflicting results highlight the need for further large-scale, randomized controlled trials to determine the precise role of IVIG in neonatal sepsis management.

GM-CSF stimulates neutrophil production, addressing the impaired immune response in neonates. While it increases neutrophil counts, multiple studies have shown no significant impact on mortality [41]. The failure of GM-CSF to improve clinical outcomes suggests that enhancing neutrophil function, rather than just quantity, may be more beneficial [41]. Emerging therapies, such as trimodulin, an innovative polyvalent antibody composition containing IgG, IgM, and IgA, show promise in activating the complement system to enhance immunity [43]. While studies in adults have been promising, there is currently no data in neonates, warranting further research into its safety and efficacy for this vulnerable population.

Vaccines

Vaccination efforts against GBS are promising strategies for preventing neonatal sepsis. As one of the main pathogens of neonatal sepsis, a GBS vaccine in conjunction with current screening guidelines would improve prenatal care. As of now, maternal vaccines targeting the GBS capsular polysaccharide are in phase 2 trials [44]. The vaccine works by inducing anti-capsular IgG antibodies in mothers [44]. This approach transfers passive immunity to neonates, reducing the need for intrapartum antibiotic prophylaxis [44]. If this trial is successful, a large burden of this preventable disease can be accounted for by enhancing maternal care. Global vaccination efforts would be the next step, ultimately avoiding neonatal infections, as many of these countries do not have robust neonatal intensive care units.

This vaccine holds promise for global implementation but faces challenges in funding, infrastructure, and acceptance. Vaccine approval requires extensive safety data for maternal-fetal use, particularly in diverse populations. Additionally, ensuring widespread acceptance and equitable distribution, especially in low-resource settings, remains a hurdle. Efforts to overcome these barriers are essential to reduce the neonatal sepsis burden worldwide.

Probiotics

Probiotics, such as strains of *Lactobacillus *and *Bifidobacterium*, are increasingly studied for their role in neonatal health. The main roles of probiotics are to help restore gut microbiome balance, promote intestinal barrier integrity, and modulate immune responses [45]. Although probiotics have not consistently reduced sepsis incidence, they provide indirect benefits to neonates, particularly in reducing necrotizing enterocolitis (NEC), a significant risk factor for sepsis in preterm infants, as seen in many studies [45, 46].

Additionally, if a neonate is on empiric antibiotic therapy to manage sepsis, a probiotic in conjunction may help maintain their gut microbiome in balance [46]. With antibiotics potentially causing dysbiosis, a probiotic may help reduce this occurrence. Yet, the exact type of probiotic strain, as hundreds exist, remains to be determined. There is significant variability in probiotic strains, dosages, and administration protocols, which has led to inconsistent results across studies [46]. Future research should focus on standardizing probiotic use and evaluating its long-term impact on neonatal immunity and sepsis prevention.

Artificial Intelligence (AI) in Neonatal Sepsis Prediction

AI predicts sepsis risk by analyzing birth weight, CRP, and maternal history, detecting patterns up to 24 hours before symptoms in pilot studies [47]. Integration with electronic health records (EHRs) could enhance monitoring, but data consistency and generalizability, especially in low-income settings, are challenges [47]. AI remains an adjunct to clinician-led care, requiring validation.

Clinical decision-making in neonatal sepsis

Effective management of neonatal sepsis hinges on standardized clinical pathways, risk assessment tools, diagnostic tests, biomarkers, and strategies to address global health disparities. This section reviews current approaches to clinical decision-making, diagnostic modalities, emerging technologies, and challenges in resource-limited settings to ensure timely and evidence-based interventions.

Clinical Pathways and Risk Assessment Tools

Clinical pathways, such as those at the Children’s Hospital of Philadelphia, integrate maternal health, prenatal care, and neonatal status to standardize sepsis management [48]. A study by Bain et al. evaluated an EOS pathway requiring neonatologist presence for risk factors (e.g., maternal fever >38°C, prolonged rupture of membranes >18 hours, inadequate GBS prophylaxis, or late preterm birth) and vital sign checks every four hours, reducing antibiotic use from 6.7% to 2.6% and CRP testing from 13.3% to 5.3% over 19 months [49]. Risk assessment tools, like the Kaiser Permanente Early-Onset Sepsis Calculator, reduce empiric antibiotic use, as shown by Achten et al. [50]. The World Health Organization (WHO)/United Nations International Children’s Emergency Fund (UNICEF) Integrated Management of Newborn and Childhood Illness (IMNCI) provides global guidelines for neonatal care, standardizing sepsis symptom recognition to facilitate diagnosis across diverse settings [51, 52].

Diagnostic Tests

Blood culture is the gold standard for diagnosing neonatal sepsis, despite low sensitivity from small sample volumes (<1 mL) or low bacterial loads [36]. Group B Streptococcus and *E. coli* cultures are 96% to 100% positive by 36 hours [53]. Gram stains, performed within hours, differentiate Gram-positive (e.g., GBS, *Listeria monocytogenes, Staphylococcus aureus*) from Gram-negative (e.g., *Escherichia *​​​​​​*coli*) organisms, guiding empiric therapy [54]. Gram-stain-specific-probe-based real-time PCR enhances detection by identifying bacterial DNA rapidly, even in culture-negative cases, as demonstrated by Wu et al. [54]. Multiplex PCR assays improve efficiency by identifying multiple pathogens, targeting GBS-specific genes [20]. Cerebrospinal fluid culture is essential due to meningitis risk, with GBS, *Escherichia coli*, *Listeria monocytogenes*, and *Staphylococcus aureus* as common causes [55]. Stoll et al. reported 38% of meningitis cases had negative blood cultures, necessitating lumbar puncture, although prior antibiotics may reduce CSF yield [16, 20]. Zhu et al. and Xu et al. identified *Escherichia *​​​​​​​*coli,* GBS, *MRSA*, and* Listeria monocytogenes* in meningitis cases, with *Listeria monocytogenes* occasionally CSF-positive but blood-negative [55, 56]. Urine cultures detect concurrent urinary tract infections (UTIs), with Kamel et al. reporting 11% urine positivity in septic neonates, more common in late-onset sepsis [57].

Biomarkers

CRP, procalcitonin (PCT), and interleukin-6 (IL-6) support diagnosis, with IL-6 aiding early suspicion [58]. The American Academy of Pediatrics (2021) recommends a full sepsis workup, including blood, urine, and CSF cultures with biomarkers, interpreted clinically to avoid overdiagnosis [59]. Next-generation sequencing (NGS) and microbial cell-free DNA (mcfDNA) sequencing offer rapid, sensitive pathogen detection, bypassing culture delays and identifying organisms despite prior antibiotics [60]. High costs and infrastructure needs limit their use, particularly in low-resource settings, where simplified protocols and funding are needed [61].

Addressing Global Health Disparities

In low- and middle-income countries, neonatal sepsis mortality exceeds 30 per 1,000 live births due to limited screening, NICU access, and trained personnel [62]. The Every Mother, Every Newborn (EMEN) initiative promotes community-based care, simplified antibiotic regimens, and training programs, reducing mortality in areas like rural Bangladesh [62]. Financial barriers, including inadequate insurance and shortages of neonatal nurses, persist, requiring funding and incentivized training to enhance care equity [62]. Scalable diagnostics, such as low-cost PCR or biomarker assays, are critical to improve timely identification in LMICs.

Currently, clinical pathways, risk assessment tools, and a combination of blood, CSF, and urine cultures, supported by Gram stains, PCR, and biomarkers, form the backbone of neonatal sepsis diagnosis. However, emerging technologies and global health initiatives are essential to address disparities and enhance outcomes, particularly in resource-limited settings.

## Conclusions

Over recent decades, the global burden of neonatal sepsis has continued to evolve, with improvements in prevention, early recognition, and supportive care contributing to better outcomes in some settings. However, LMICs face disproportionately high mortality due to limited neonatal specialty care and economic barriers. Standardized, universal guidelines are essential to ensure equitable diagnosis and management worldwide. While blood culture remains the diagnostic cornerstone, emerging technologies such as next-generation sequencing offer enhanced detection potential; however, their high cost and infrastructure requirements currently limit widespread implementation in resource-constrained settings. Evidence-based algorithms that integrate clinical pathways and risk assessment tools can drive consistent, timely interventions. Maternal GBS vaccines, novel immunotherapies, and AI-driven predictive models hold transformative promise to reduce the neonatal sepsis burden. Global initiatives must prioritize scalable screening, prevention, and treatment strategies, supported by increased funding and training, especially in underserved regions. A unified global commitment to implement these innovations is critical to protect vulnerable newborns and eliminate preventable deaths from neonatal sepsis.
